# Implementation and impact of rapid SARS-CoV-2 point of care test on patient flow in the frailty pathway; A quality improvement approach

**DOI:** 10.1371/journal.pone.0296294

**Published:** 2024-01-02

**Authors:** Ijeoma Okoliegbe, Louise Brodie, Alistair Cowie, Brandon Tan, Suzanne Clements, Zoe Muir, Noha El Sakka

**Affiliations:** 1 School of Medicine, Medical Sciences and Nutrition, University of Aberdeen, Aberdeen, Scotland, United Kingdom; 2 Department of Medical Microbiology and Virology, Aberdeen Royal Infirmary, Aberdeen, Scotland, United Kingdom; 3 Department of Medicine for the elderly, Aberdeen Royal Infirmary Aberdeen, Aberdeen, Scotland, United Kingdom; All India Institute of Medical Sciences, INDIA

## Abstract

To maintain patient flow during the COVID-19 pandemic, rapid and accurate decisions for the safe triage of geriatrics patients was essential as turnaround times for laboratory testing was ineffective at supporting rapid clinical decision-making for transfer of care. Thus, to mitigate and inform these clinical decisions, a quality improvement collaborative project with the geriatrics and virology department was conducted at the Frailty Assessment Unit (FAU) at Aberdeen Royal Infirmary. The goal was to facilitate patient triage during transfer of care with the introduction of Point of Care testing (POCT). The interventions which resulted in significant improvements were based on the fishbone problem solving approach and the driver diagram with change ideas informing the five Plan, Do, Study and Act (PDSA) cycles. The QI intervention was crucial in supporting clinical staff decision making during transfers for 95% of patients who had been clinically judged as asymptomatic for COVID-19 infection. High staff engagement was observed with 83% of staff suggesting the process map was easy to follow and 92% of clinical staff agreed it contained sufficient information to support the testing process. With POCT introduction, the proportion of patients who were transferred with an early POCT result increased by 20% in the Rosewell House group and by 65% in the community Hospitals group, once governance arrangements were in place. Finally, the considerable uptake of POCT by the ward consequently led to a decrease of up to 86% in the number of samples sent to the laboratory for rapid SARS-CoV-2 testing. The quality improvement project provided a rapid and reliable SARS-CoV-2 triage tool and was effectively integrated into the geriatrics triage algorithm to facilitate patient placement and flow.

## Problem

During COVID-19 pandemic, the emergence and rapid spread of severe acute respiratory syndrome coronavirus-2 (SARS-CoV-2) represented a challenge for clinical wards and laboratories. Clinical wards including the geriatrics department relied on timely and reliable diagnostic information to maintain its patient flow while providing safe and high-quality care. In clinical practice there was a need for quick turnaround time for SARS-CoV-2 results to aid rapid clinical decision making, particularly in relation to patient placement especially in the early implementation of infection control measures and maintaining patient flow through discharges from hospital. Additionally, the national guidance on asymptomatic SARS-CoV-2 testing required testing on admission, on day 5 of in-patient stay and 48hr pre-discharge or transfer. This further compounded patient flow in the frailty pathway. The laboratory responded to the pandemic by routine batch testing using 96-well plates. This approach increased the number of samples tested and reduced staffing requirements but did not aid the clinicians for rapid decision making in the wards. To help improve timely patient discharges and patient flow in the wards, the laboratory introduced an additional testing pathway which enabled wards to send requests for rapid SARS-CoV-2 testing with shorter turnaround time (TAT) for time-critical results. In this pathway, three instruments manned by staff were set up in the laboratory for rapid SARS-CoV-2 testing. Although this potentially reduced the TAT, the laboratory observed that this pathway had a low sample throughput due to the number of samples which could be tested at one time on each instrument as well as staffing demand. One staff member can process only 1 sample at a given time. Additionally, with the establishment of a new testing pathway to help with the newly introduced admission and discharge testing requirements, the laboratory experienced an unusually high number of requests for rapid testing out-with routine batch testing. This created a burden on laboratory staff as more staff time was required, which affected routine provisions of laboratory services. Given the importance of an efficient system for SARS-CoV-2 testing, our aim was to improve patient flow in the frailty assessment unit and to rationalise the number of requests for the laboratory’s newly introduced rapid testing pathway.

## Background

Coronavirus disease 2019 (COVID-19) infection which has rapidly spread globally causes a respiratory illness, transmitted majorly through respiratory droplets and direct contact [1]. During the pandemic, data demonstrated that the older adults, patients with multiple comorbidities were particularly susceptible to this infection with increased mortality compared to the younger population. These observations are mirrored in the clinical outcome of other respiratory viral infections such as seasonal Influenza and SARS (severe acute respiratory syndrome) [2–4]. As the pandemic progressed, it was imperative that providing rapid diagnosis of COVID-19 cases was crucial in maintaining and optimizing patient care in hospitals. In addition, rapid testing was also important to help timely decision-making for patient placement and transfers, and to prevent nosocomial infections within the healthcare setting [5–8].

Real-time PCR is the gold standard for diagnostic respiratory virus testing. However, with few patient isolation facilities, rising waves of variants of concern, and the urgent need for bed allocation, patient flow and cohorting, several point of care testing technologies has been employed at front door admission and discharge facilities [9, 10].

We employed the simple user-friendly Abbott ID NOW COVID-19 molecular assay, to enhance patient flow in the frailty assessment unit. The assay uses isothermal nucleic acid technology to amplify a unique region of the RNA-dependent RNA polymerase (RdRp) viral gene which is detected using fluorescently labelled molecular beacons. It has a TAT of 5–13 minutes, which is considerably shorter than the lab based real-time PCR assay. This has the potential of promptly informing clinical decisions for transfers and discharge.

## Measurement

To gain a snapshot on the impact of TAT for testing on the service provision, baseline data collection focused on discharges patients in the frailty assessment unit of Aberdeen Royal Infirmary for three days during a single working week. We measured the pre-analytical TAT (collection and transport to the laboratory) and the analytical TAT (laboratory processing to results), for 33 patients requiring transfer from the geriatrics department to other facilities (S1 Fig). This data identified that despite 27% (9/33) of patients being symptomatic the median TAT was 891 mins from sample collection to results. In addition, it demonstrated that 82.60% (736/891 minutes) of the total TAT was composed of the analytical TAT. Furthermore, varying differences in the pre-analytical TAT were observed and this markedly impacted on COVID-19 result. The pre-analytical and analytical TAT highlighted the urgent need for quality improvement in this pathway. A fish-bone cause and effect exercise were carried out to identify possible causes of the problem and to group ideas into useful categories to explore in achieving the aim.

## Design

**Fig 1:**
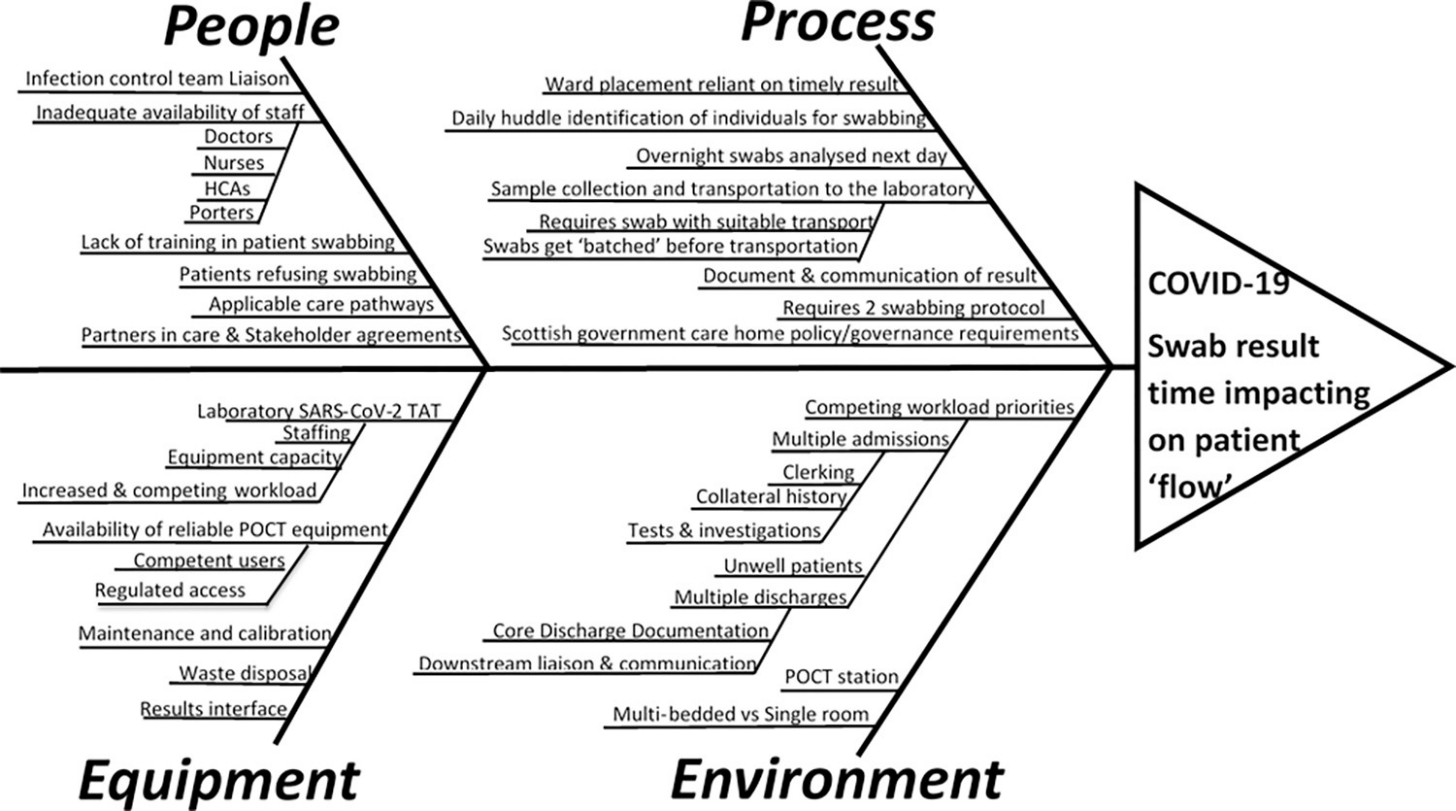
A fishbone diagram illustrating the major obstacles to early SARS-CoV-2 test results in the frailty ward.

Increased SARS-CoV-2 test TAT had a negative impacting on patient flow at the frailty assessment unit, which required exploring areas for improvement. A quality improvement (QI) team consisting of key stakeholders including a clinician champion, QI officer, point of care testing (POCT) champion, clinical staff and representatives from the virology laboratory was formed. The QI team used a fishbone diagram as a problem-solving approach to identify the root causes of increased TAT and troubleshoot possible solutions (Fig 1). The team explored causes affecting the SARS-CoV-2 testing process such as batch transportation of swabs and considered the existing policy and governance requirements. Additionally, ward and staff associated factors as well as POCT equipment considerations were also considered by the team.

**Fig 2:**
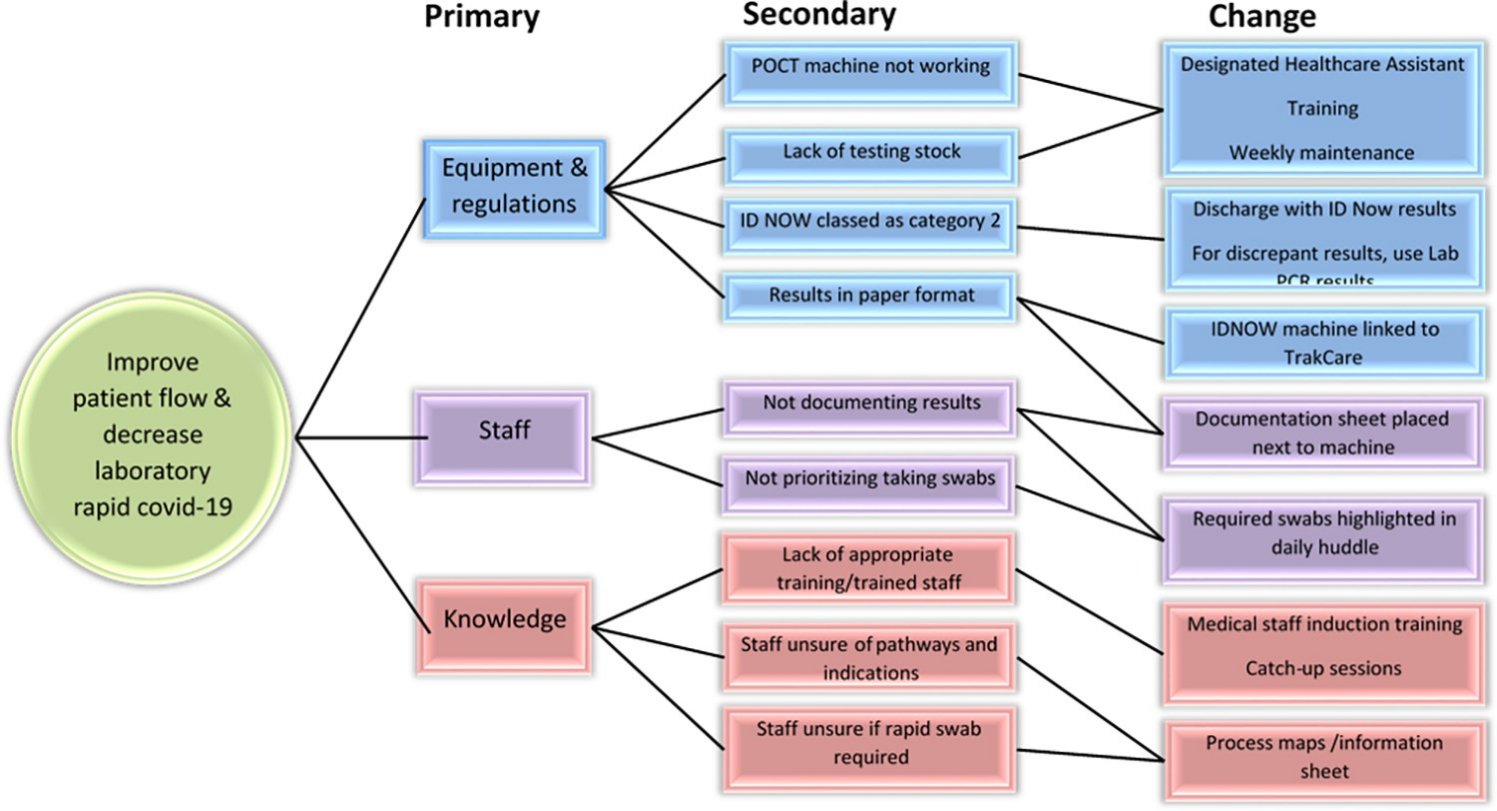
Driver diagram and change ideas implemented during the quality improvement intervention.

Consequently, a driver diagram (Fig 2) was used with primary drivers such as staff, knowledge, equipment and regulations further delineated into change ideas. The initial aim was to improve the TAT for SARS-CoV-2 tests in this patient population. This outcome was crucial in the management of patients and in maintaining flow in the ward. A secondary outcome was to reduce the number of samples sent for rapid SARS-CoV-2 testing in the laboratory, and instead allow the rapid testing to be performed in the ward as POCT.

The QI project took place at the Frailty Assessment Unit of the Aberdeen Royal Infirmary, Aberdeen City, Scotland, UK and the Rosewell House, which is an offsite stepdown ward run by Aberdeen City Health and Social Care Partnership. As both sites were in Aberdeen city, they shared a similar governance arrangement. The learnings from this QI were used to inform the scaling up of discharges to the Community Hospitals which was managed by the Aberdeenshire Health and Social Care Partnership and had a different governance arrangement.

All patients in the unit who are ready for transfer to Rosewell House and latterly the Community Hospitals were tested in the ward using POCT. Initially, to ensure there was no discrepancy with PCR testing during the QI and to build confidence in all transitions of care from the frailty ward, a second sample was sent to the laboratory to be tested for SARS-CoV-2 PCR as per standard of care. POCT was carried out using a mid-turbinate dry nasal swab and testing was carried out by the clinical staff using the Abbott IDNOW instrument while paired combined nose and throat sample for RT-PCR testing was sent to the virology laboratory to be processed using either the Seegene or Altona assays. To improve TAT and ease of testing, a POCT equipment (IDNOW) was placed in the geriatric unit. A healthcare worker in the ward was assigned as the ward POCT champion with duties involving equipment maintenance and stock availability. Training was provided for staff to support knowledge, documentation, testing pathways and indications by the company representative and the virology staff while continuing education was carried out by the ward champion and cascade trainer. As the geriatrics ward has a high staff turnover rate, training was designed to coincide with the induction week of doctor’s rotation in February, April, August and December. Staff competency was assessed by the ward champion and the virology laboratory staff. For traceability and documentation, all results from the POCT equipment were electronically transferred to the electronic patient records the same way as with the laboratory RT-PCR results with relevant public health notifications. To allow differentiation from routine testing, POCT samples were assigned a sample code different from the virology laboratory code.

## Strategy

**Fig 3:**
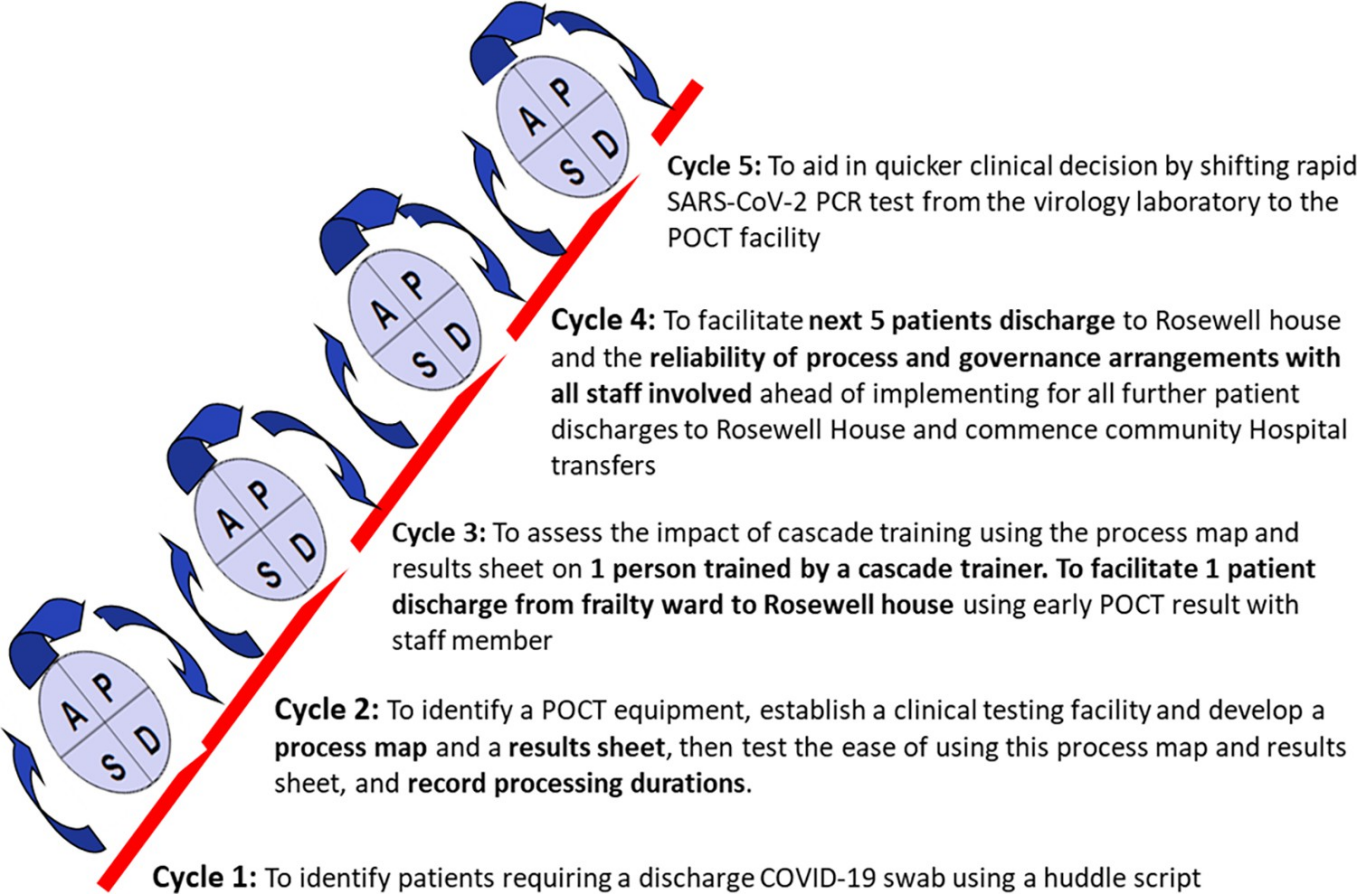
Plan, Do, Study and Act cycles carried out during the quality improvement intervention.

This quality improvement project was carried out between June 2021 and September 2022. Our SMART (Specific, Measurable, Achievable, Relevant and Time-bound) aim was to improve patient flow through reducing SARS-CoV-2 TAT and to shift the bulk of rapid SARS-CoV-2 testing from being sent to the virology lab to being tested in the ward by POCT. We undertook five PDSA (Plan, Do, Study and Act) test cycles (Fig 3) which are summarized below.

### PDSA cycle 1

The initial intervention was to develop a system of identifying patients who required a discharge COVID-19 swab before transfer. The daily afternoon huddle was explored as the suitable time to identify these patients. Thus, the QI team created a huddle script with a check, chase and challenge task list (S2 Fig). This script ensured staff focused on tasks, identified discharge plans due in the next 12-24hrs and assessed if COVID-19 swabs were in-date for discharge. A secondary outcome was to assess the impact this intervention had on huddle duration and evaluate if it resulted in unnecessary swabs for patients. Measurement was assessed by identifying the number of patients who had been delayed due to a COVID-19 swab result. In addition, feedback was collected from the nursing staff and the discharge coordinator on the impact of COVID-19 results on discharge plans. To ensure information was accessible to every member of staff, the ward’s whiteboard was updated with the date and results of the last COVID-19 swab for all patients before the huddle. A column was added to the whiteboard to show when discharge swabs were taken for transfer of care. Over the course of 1 week (22nd –29th April 2021) a total of 25 patients were included in this cycle. This PDSA cycle concluded at evaluation and the agreement of the QI team to adopt and embed this as best practice for all patients admitted to the ward necessitating transfer of care to alternative clinical environments ahead of discharge. Feedback from the discharge coordinators highlighted that this intervention was a helpful prompt to all staff of the requirement to obtain swabs within the agreed pre transition of care window, however there was still room for improvement in the time taken to obtain swab results to enable the transfer of the patient to progress.

### PDSA cycle 2

An IDNOW POCT equipment was identified, and a testing station was established in the Frailty Assessment Unit (S3 Fig). The effectiveness of using a POCT in determining COVID-19 status and its impact in supporting clinical decisions was explored. The cycle assessed the length of time from patient arrival to sample collection and processing, the testing process and its ease of use, the need for guides and reminders on testing and the best way to record and handover COVID-19 results. A crucial learning in this cycle is the verbose nature of the initial process map. The QI team considered that reduction in instructions might lead to loss of essential information during testing. To enhance staff clarity the process map was redesigned as a flow chart while colours were used to highlight key events (S4 Fig). Instructions to check the results of the laboratory SARS-CoV-2 PCR test were included at the end of the process map. The designated doctor in charge of discharges was assigned to check this task. Prior to IT interfacing, an email and phone call alert system were implemented for new COVID-19-positive PCR results to virology department to enter the results on the laboratory information management system which triggered public health notifications for COVID-19. The ward also communicated these results to the Infection Control team which facilitated rapid alerting of patients requiring transfer for COVID-19 isolation and contact precautions by clinical staff. This requirement was stopped once IT interphase was put in place. The Frailty Assessment Unit has a high staff turnover with annual rotations every February, August, and December. To ensure testing continuity and training, staff members were trained to become cascade trainers, and training support was offered during the annual rotations by the virology department.

### PDSA cycle 3

To build and strengthen the testing and training process, a patient journey was studied for a clinical staff who had been trained by a cascade trainer. It was observed that due to the multidisciplinary nature of healthcare, other health professionals and patient characteristics might impact on sample collection with durations of >10 minutes observed. This inadvertently affected testing as instrument timeout was observed while following the manufacturer’s instructions. To avoid instrument timeout, machine preparation was placed after sample collection in the process map. POCT samples were collected at the patient’s bedside with swabs re-sheathed for transport to the testing station, and staff expressed concerns about samples and environmental contamination. The process map was updated to include specimen bags for sample collection with a specimen tray placed beside the testing equipment. Learning from this cycle informed the final process map (S4 Fig). The QI team produced testing guides and infographics to support training. Similarly, competency documents were developed to assess learning and to support the cascade trainers during training. The cascade trainers also encouraged staff to use the instructional videos to support their learning (https://www.globalpointofcare.abbott/en/support/product-installation-training/id-now-training-videos.html). Additionally, for quality and traceability, a restricted machine access for user management emphasized that only trained and competent staff could be assigned logins after competency assessment by the cascade trainers.

### PDSA cycle 4

The intervention was expanded to include 5 patient journeys to Rosewell House from the Frailty ward and study how it would improve patient flow. These facilities are situated in Aberdeen city and share similar governance processes as patients could be transferred to each facility without the gold standard PCR testing. Afterwards, a one-month survey was conducted to assess the impact of the intervention on TATs for 41 patients (S5 Fig). With a 98% reduction in TATs informing clinical decisions, POCT was expanded to include all patients for Rosewell House transfers. The learnings from Rosewell House informed the practice in community hospitals transfer. Additionally, with the expansion of testing, a crucial part of this intervention was feedback from staff who were involved in the testing process. Using a qualitative approach, random responses from 12 members of staff were used to assess the impact of the change idea and staff perception.

### PDSA cycle 5

We hypothesized the change idea would be effective in shifting the request for rapid SARS-CoV-2 testing by the unit from the virology laboratory to the POCT facility in the ward to enable it to inform clinical decisions. Six months post introduction of POCT, the proportion of samples sent by the ward to the laboratory for rapid testing was assessed.

## Results

The QI project targeted the frailty pathway with a population comprising elderly patients (mean, 85±7 years) (Table 1). Only 4.97% (n = 9/18 patients) were symptomatic of COVID-19 of which 88% (n = 8/9 patients) were diagnosed with the infection highlighting the efficiency of the clinical tool for diagnosis. However, with the asymptomatic transmission of SARS-CoV-2 during the pandemic, clinical staff required support in identifying positive SARS-CoV-2 patients to aid in surveillance and infection control. This QI intervention was used to aid clinical staff in decision making for 95% (n = 172/181) of patients who had been clinically judged as asymptomatic for COVID-19 infection highlighting its role in patient care and flow.

**Table 1:** Patient demographics.

		Rosewell House (%)	Community Hospital (%)	All patients (%)
Number of patients		106 (58.56)	75 (41.44)	181 (100.00)
Gender	Male	38 (35.85)	28 (37.33)	66 (36.46)
	Female	68 (64.15)	47 (62.67)	115 (63.54)
Age (mean)		86 ± 6.3	84 ± 7.5	85 ± 6.8
COVID-19 symptoms				
Asymptomatic		100 (94.34)	72	172 (95.03)
Symptomatic		6 (5.66)	3 (4.00)	9 (4.97)
COVID-19 positive		6 (5.66)	2 (2.67)	8 (4.42)

The QI project was well-received by the clinical team as it supported staff when making decisions on patient placement and flow (Table 2). Also, its impact on family visits and transfer to an appropriate care facility was highlighted.

**Table 2:** Qualitative feedback of POCT for patients and staff.

Staff	What benefit does POCT deliver for patients?	What benefit does POCT deliver for staff?
S01	Timely transition of care alongside positioning of patients in multi-bedded rooms at the earliest opportunity.	Confidence in the COVID-19 status. Patient transition no longer delayed because of awaiting this outcome
S02	Faster management plan making	Able to quickly ascertain COVID status rather than waiting for lab swab report
S03	Useful for discharge planning	Easy to use, quick results
S04	Allows patients to be moved from single-bedded rooms to multi bedded rooms more quickly, allowing patients to get monitored more 1–1. Allows patients families to come and visit more timely	Allows patients to be moved from single-bedded rooms to multi-bedded rooms more quickly, which helps allow for more beds.
S05	It’s helpful with high falls risk patients to have a negative result when exploring the corridors and so we can move them to a multi-bedded room	Easier moving patients around the ward
S06	Help the flow of patients in terms of moving to bay bed, boarding, discharge and save the time and cost of using visors for every patient and improve the communication with patients with reduced hearing without visors as well	It saves time required for planning of discharges and assure staff when patients are negative, so they feel safer while doing their assessment
S07	Faster COVID-19 result	Faster result for patient flow
S08	No response	
S09	Reduced delay in getting to appropriate care facility	Enables quick identification of COVID-19 and reduces exposure
S10	Quicker test result	Faster result
S11	No response	
S12	Quicker turnaround	Quicker turnaround

**Fig 4:**
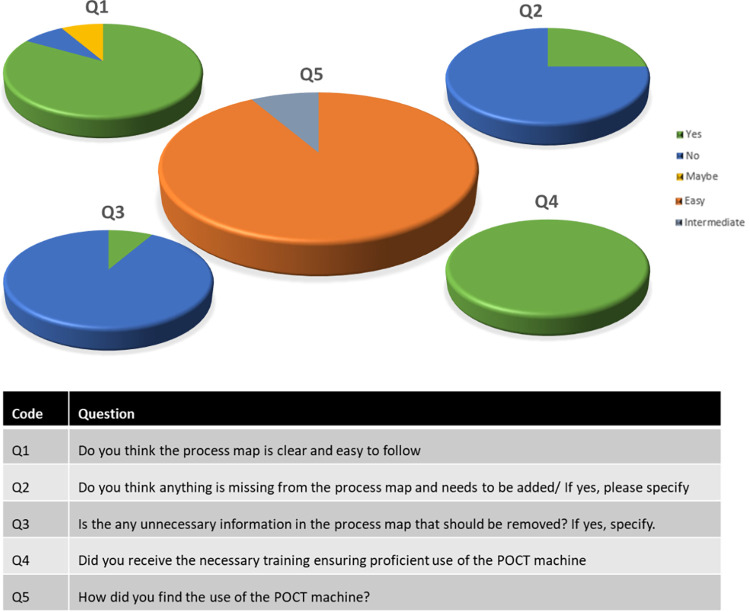
Qualitative analysis of staff views on the process map and POCT equipment.

Using a qualitative approach for staff feedback, all staff members received appropriate training in the efficient use of the POCT equipment (Fig 4). Of these, 92% (n = 11/12) reported the POCT equipment was easy to use. Furthermore, 83% (n = 10/12) suggested the process map was easy to follow and 92% (n = 11/12) agreed that the information contained in the map was adequate to support the testing process.

**Fig 5:**
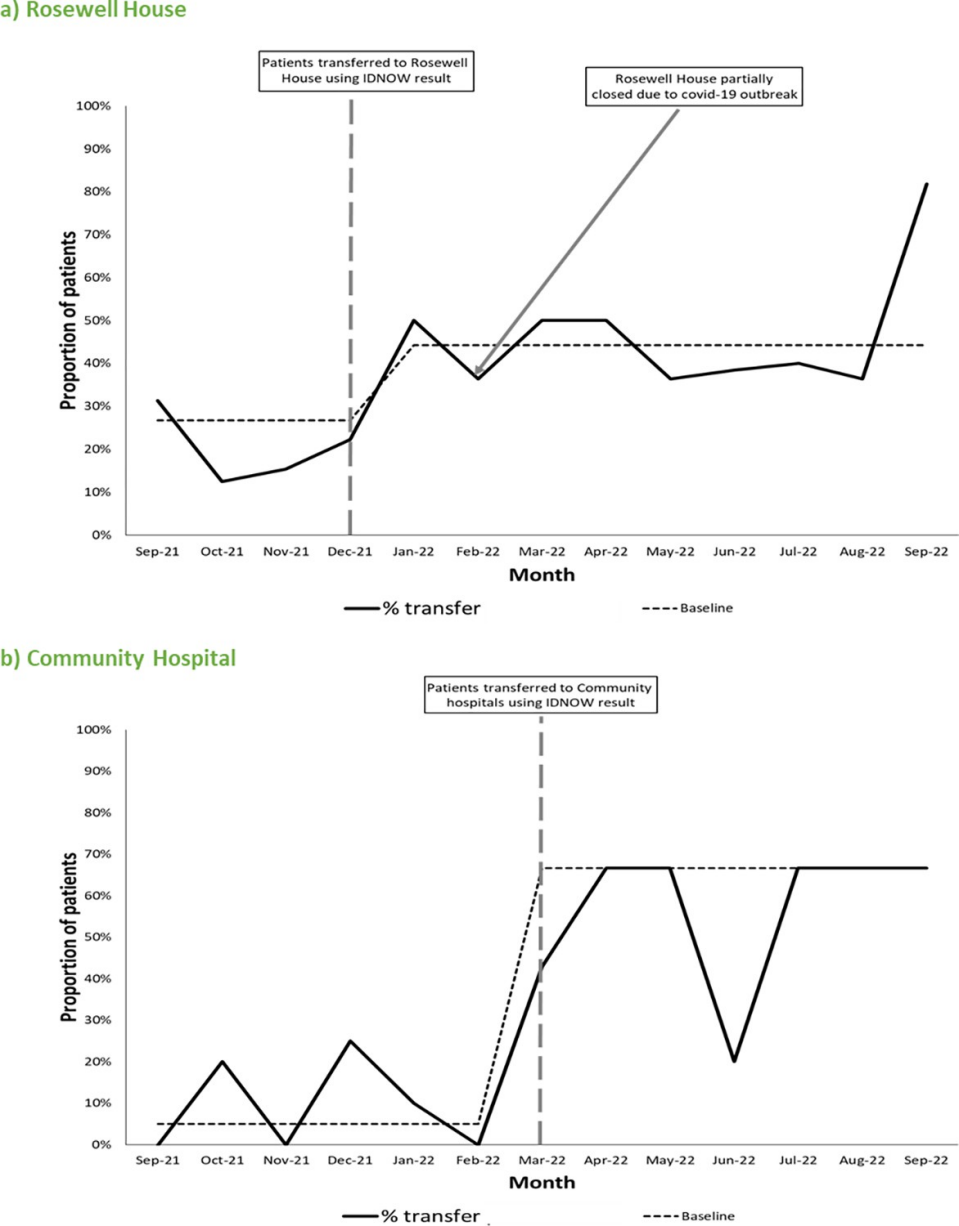
Monthly proportion of patients transferred to a) Rosewell House and b) Community Hospitals with an early Abbott ID Now SARS-CoV-2 results. Overall median was used to calculate the baseline was described by Clarke et al 2009 [11].

Using a time-series analyses, the proportion of patients who were transferred with an early POCT result increased by 20% in the Rosewell House group and by 65% in the community Hospitals group, once governance arrangements were in place (Fig 5). Interestingly, data collected from POCT introduction (Oct 2021) until first rotation (Dec 2021) demonstrated an upward increase in transfer of patients to Rosewell House and growing acceptance of the testing strategy by staff members. This growing acceptance by staff was highlighted in Feb 2022 which showed that despite the partial closure of Rosewell House due to an outbreak, there was only a 29% decrease in patient transfers to the care facility (Fig 5).

**Fig 6:**
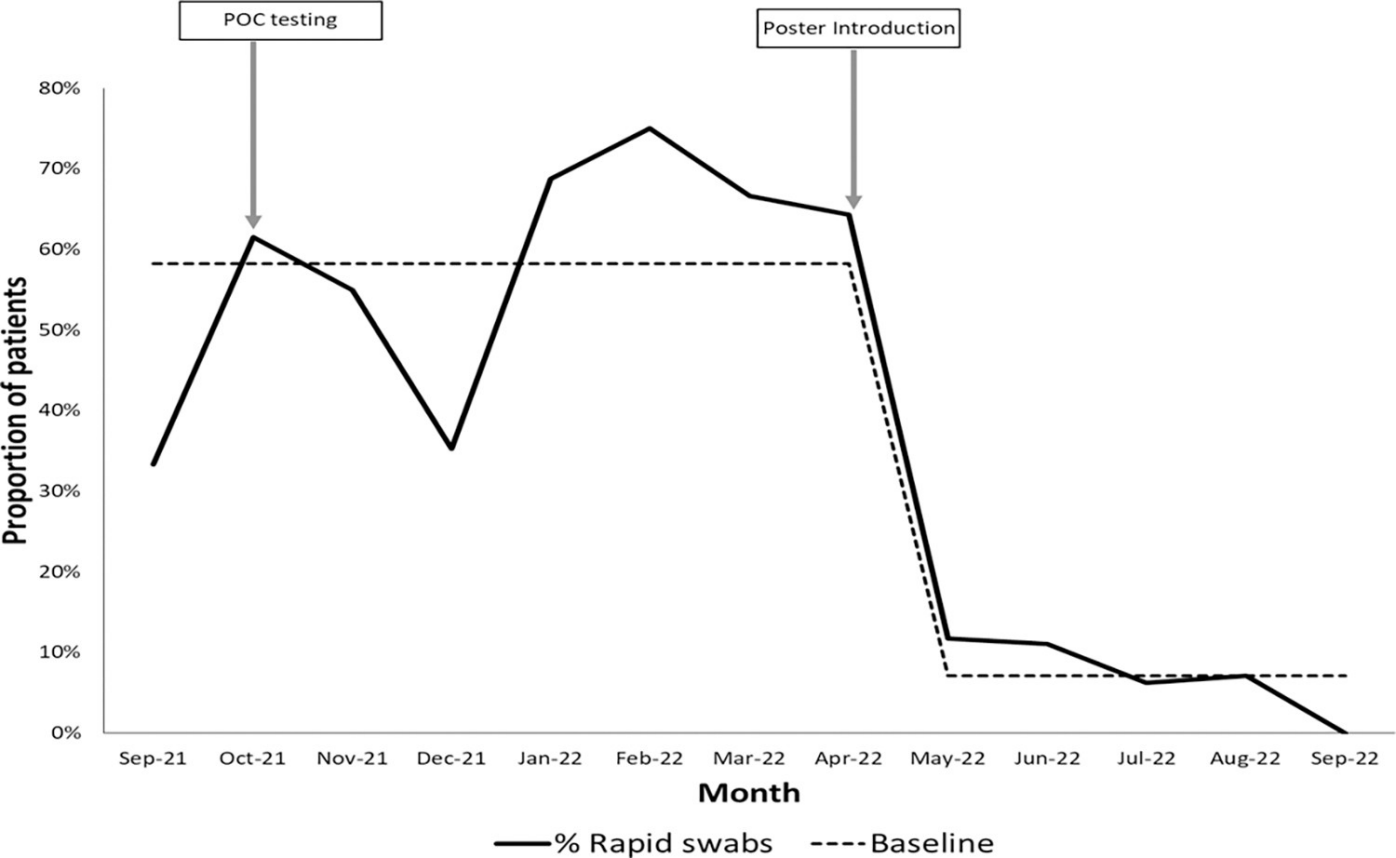
Time-series describing the monthly proportion of patients with rapid swabs during the quality improvement project. Overall median was used to calculate the baseline was described by Clarke et al 2009 [11].

The QI intervention led to a considerable uptake of POCT by the ward and consequently a decrease in the number of samples sent to the laboratory for rapid SARS-CoV-2 testing (Fig 6). However, with general testing fatigue among staff from Jan 2022, more demonstration tools were needed to highlight this testing strategy to the attention of staff. The QI team introduced a poster to aid staff during decision making (S6 Fig). An 86% decrease in the number of rapid SARS-CoV-2 tests sent by the ward to the virology laboratory was observed when the poster was introduced in May 2022 (Fig 6).

## Lessons and limitations

Our findings suggest that the use of effective and simple reminders during the daily huddles can have a positive impact, at least in the short term, in identifying patients who were due a COVID-19 discharge swab. The intervention that proved most effective involved the editable “whiteboard” which was quick and simple to complete. The use of this patient-centered white board was effective as it is already a part of the doctors/nurses’ workflow. Using prompts and reminders before each huddle ensured there was a central view for outstanding tasks and a promotion of teamwork and effective communication. This approach had a greater chance of long-term sustainability. Similarly, the use of several means of communication for training and testing was successful in steering the success of this project. Indeed, the consideration for human factors with the colour-coding of the process map was crucial in reducing ambiguity during testing. All these factors better supported staff who experienced the impact of early COVID-19 result in patient management and were enthused to share skills through cascade training ensuring business continuity for COVID-19 testing. Additionally, training support by the virology laboratory at the induction week of each staff rotation guaranteed there was testing continuity in a ward which experienced a high staff turnaround. The QI project benefitted from the support of the cascade trainers who maintained training requirements during the intervention. Equally, the designation of a ward POCT champion who oversaw staff training and competency assessment, user management, kits supply, weekly quality controls and equipment maintenance immensely contributed to the QI success. But, ensuring compliance and adherence to the process map alongside the governance arrangements of the partnership on the established pathway was initially difficult. Staff frequently required the reinforcement of safe disposal guidance and documentation of subjects for sample correlation purposes Finally, the use of the short feedback questionnaire while assessing the impact of early POCT results on discharge documentation and testing ensured staff were enthusiastic in co-producing the QI intervention.

The aforementioned factors contributed and led to an improvement in patient discharge from the frailty ward to Rosewell House and latterly to the Community Hospitals (S7 Fig). However, it is not possible to conclude that the early POCT COVID-19 results had a direct impact on patient flow as discharge plans are multi-factorial but testing TAT was one of the main factors that impacted the process. There is evidence that improving the TAT of testing as one of the main stems in the patient journey has contributed to an overall improvement of patient flow and discharge in the ward [12, 13].

However, the project was limited by several factors. Initially the limitation encountered was because the ward was only able to transition patients to the Rosewell House facility using the IDNOW outcome as this facility was managed by Aberdeen City Health and social care partnership, who also managed the GAU and were responsible for the governance arrangements. This meant all other transfers of care out with Rosewell House had to await the return of a specimen outcome from the laboratory which took longer to result. This inadvertently affected transport arrangements which could then only be booked with the ambulance team on receipt of COVID-19 result. Also, care homes across Aberdeen City and Aberdeenshire would only accept results that were provided from laboratory specimens in line with government legislation. Results from this Quality Improvement project ensured we were able to extend POCT to these pathways. Finally, another limiting factor in our observation was the inability to account for length of stay for patients in the wards and its consideration as baseline measurement. Other factors such as comorbidity, clinical severity of illness, functional status or age have been demonstrated to impact on patient discharges and transfers [14, 15].

## Conclusion

The key focus of all the interventions in this QI project was to ensure efficient and continued implementation of a sustainable SARS-CoV-2 screening solution rather than a short-term quick-fix in the geriatric’s population in our board. The QI team members ensured participation of all stakeholders using a co-production approach in communicating learnings with clear messages to all staff. Additionally, this approach helped the QI team when devising new interventions based on suggestions and learnings from each intervention which supported staff engagement.

The QI project was successful in reducing rapid SARS-CoV-2 test request to the laboratory by the ward staff, and instead shifted rapid testing to POCT at the ward and improving patient flow in the Rosewell House. Nevertheless, the authors acknowledge that more work is required to explore other factors impacting on TAT for SARS-CoV-2 testing as well as to ensure the observed improvements are sustained. The QI team aims to consolidate on learnings and use the multidisciplinary approach supported by QI methodology to proactively study any deviation especially in the winter periods.

## Supporting information

S1 Fig Snapshot survey of 33 SARS-CoV-2 turnaround times in June 2021. (TIF)

S2 Fig Huddle script for identification of patients requiring COVID-19 results for transfer. (TIF)

S3 Fig Point of care testing facility set up at the frailty ward. (TIF)

S4 Fig Process map for SARS-CoV-2 testing process. (TIF)

S5 Fig One month survey comparing Lab PCR and Abbott ID Now SARS-CoV-2 turnaround times Oct 2021. (TIF)

S6 Fig Point of care testing decision aiding tool. (TIF)

S7 Fig Feedback from the acute geriatrics medicine learning from excellence team. (TIF)

## References

[1] Harapan H, Itoh N, Yufika A, Winardi W, Keam S, Te H, et al. Coronavirus disease 2019.(COVID-19): A literature review. Journal of infection and public health 2020; 13(5):667–673. 32340833 10.1016/j.jiph.2020.03.019 PMC7142680

[2] Divo MJ, Martinez CH, Mannino DM. Ageing and the epidemiology of multimorbidity. European Respiratory Journal 2014; 44(4):1055–1068. doi: 10.1183/09031936.00059814 25142482 PMC4918092

[3] Mertz D, Kim TH, Johnstone J, Lam P, Kuster SP, Fadel SA, et al. Populations at risk for severe or complicated influenza illness: systematic review and meta-analysis. BMJ 2013; 347. doi: 10.1136/bmj.f5061 23974637 PMC3805492

[4] Walker TA, Waite B, Thompson MG, McArthur C, Wong C, Baker MG, et al. Risk of severe influenza among adults with chronic medical conditions. J Infect Dis 2020; 221(2):183–190. doi: 10.1093/infdis/jiz570 31678990

[5] Kortuem SO, Krause M, Ott H, Kortuem L, Schlaudt H. Molecular point-of-care testing for SARS-CoV-2 using the ID NOW (TM) System in Emergency Department: Prospective Evaluation and Implementation in the Care Process. MedRxiv 2021.

[6] Kortüm S, Becker D, Ott H, Schlaudt HP. The role of the emergency department in protecting the hospital as a critical infrastructure in the corona pandemic strategies and experiences of a rural sole acute-care clinic. medRxiv 2020.

[7] NguyenVan J, Gerlier C, Pilmis B, Mizrahi A, de Ponfilly GP, Khaterchi A, et al. Prospective evaluation of ID NOW COVID-19 assay used as point-of-care test in an emergency department. Journal of Clinical Virology 2021; 145:105021. doi: 10.1016/j.jcv.2021.105021 34768231 PMC8556064

[8] Collier DA, Assennato SM, Warne B, Sithole N, Sharrocks K, Ritchie A, et al. Point of care nucleic acid testing for SARS-CoV-2 in hospitalized patients: a clinical validation trial and implementation study. Cell Reports Medicine 2020; 1(5):100062. doi: 10.1016/j.xcrm.2020.100062 32838340 PMC7362826

[9] Rhoads DD, Cherian SS, Roman K, Stempak LM, Schmotzer CL, Sadri N. Comparison of Abbott ID Now, DiaSorin Simplexa, and CDC FDA emergency use authorization methods for the detection of SARS-CoV-2 from nasopharyngeal and nasal swabs from individuals diagnosed with COVID-19. J Clin Microbiol 2020; 58(8):760. doi: 10.1128/JCM.00760-20 32303564 PMC7383529

[10] Harrington A, Cox B, Snowdon J, Bakst J, Ley E, Grajales P, et al. Comparison of Abbott ID Now and Abbott m2000 methods for the detection of SARS-CoV-2 from nasopharyngeal and nasal swabs from symptomatic patients. J Clin Microbiol 2020; 58(8):798. doi: 10.1128/JCM.00798-20 32327448 PMC7383519

[11] Clarke J, Davidge M, James L. The how-to guide for measurement for improvement. Patient Safety First. 2009.

[12] Ong MS, Magrabi F, Coiera E. Delay in reviewing test results prolongs hospital length of stay: a retrospective cohort study. BMC health services research. 2018; 18:1–8. 29769074 10.1186/s12913-018-3181-z PMC5956538

[13] Mustafa A, Mahgoub S. Understanding and overcoming barriers to timely discharge from the pediatric units. BMJ Open Quality. 2016 Sep 1;5(1):u209098–w3772. doi: 10.1136/bmjquality.u209098.w3772 27752313 PMC5051419

[14] van der Laag PJ, Arends SA, Bosma MS, van den Hoogen A. Factors associated with successful rehabilitation in older adults: A systematic review and best evidence synthesis. Geriatr Nurs 2021; 42(1):83–93. doi: 10.1016/j.gerinurse.2020.11.010 33387828

[15] Everink IH, van Haastregt J, van Hoof SJ, Schols JM, Kempen GI. Factors influencing home discharge after inpatient rehabilitation of older patients: a systematic review. BMC geriatrics 2016; 16(1):1–14. doi: 10.1186/s12877-016-0187-4 26755206 PMC4709872

