# Supplementary figures and images for: Implementation and impact of rapid SARS-CoV-2 point of care test on patient flow in the frailty pathway; A quality improvement approach
Source: PLoS One. 2024 Jan 2;19(1):e0296294. doi: 10.1371/journal.pone.0296294 (PMC10760674; doi:10.1371/journal.pone.0296294)

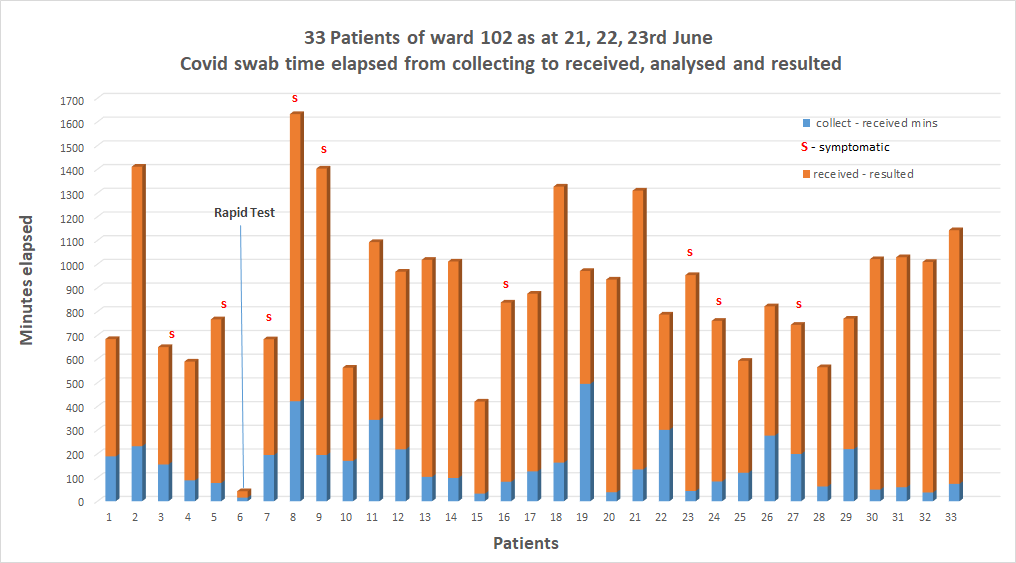

Supplement: S1 Fig — (TIF) [file pone.0296294.s001.tif]

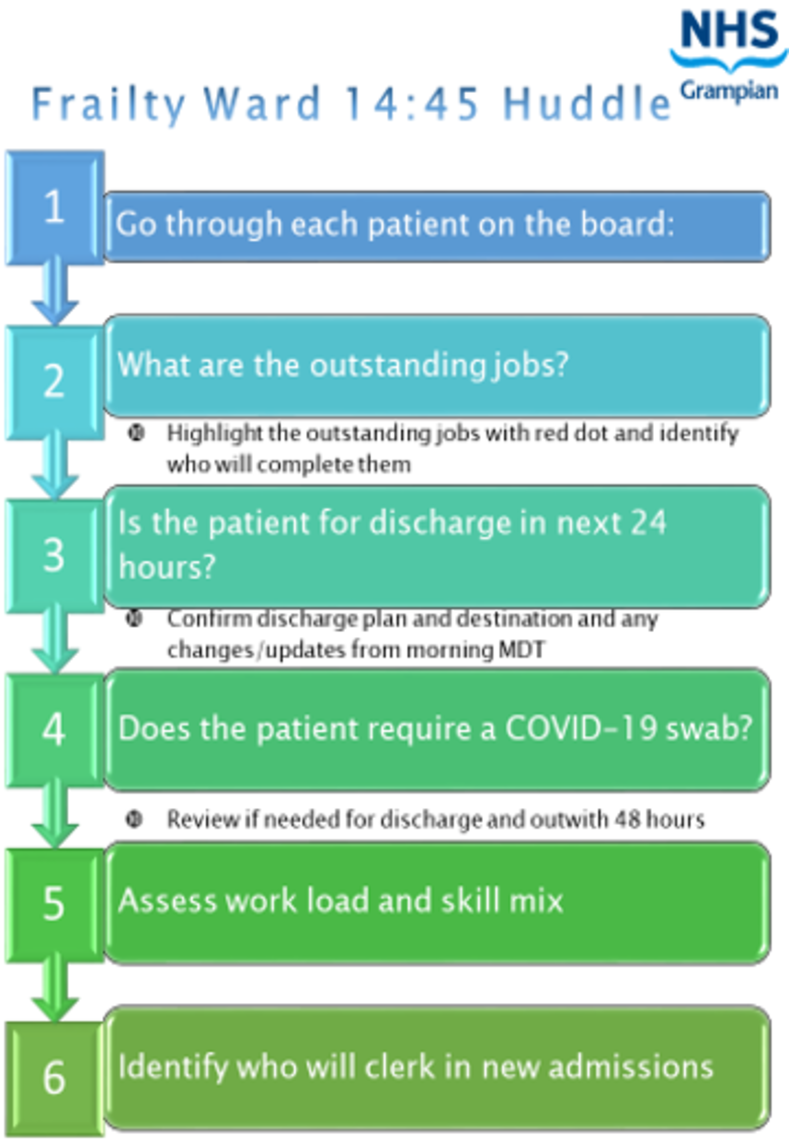

Supplement: S2 Fig — (TIF) [file pone.0296294.s002.tif]

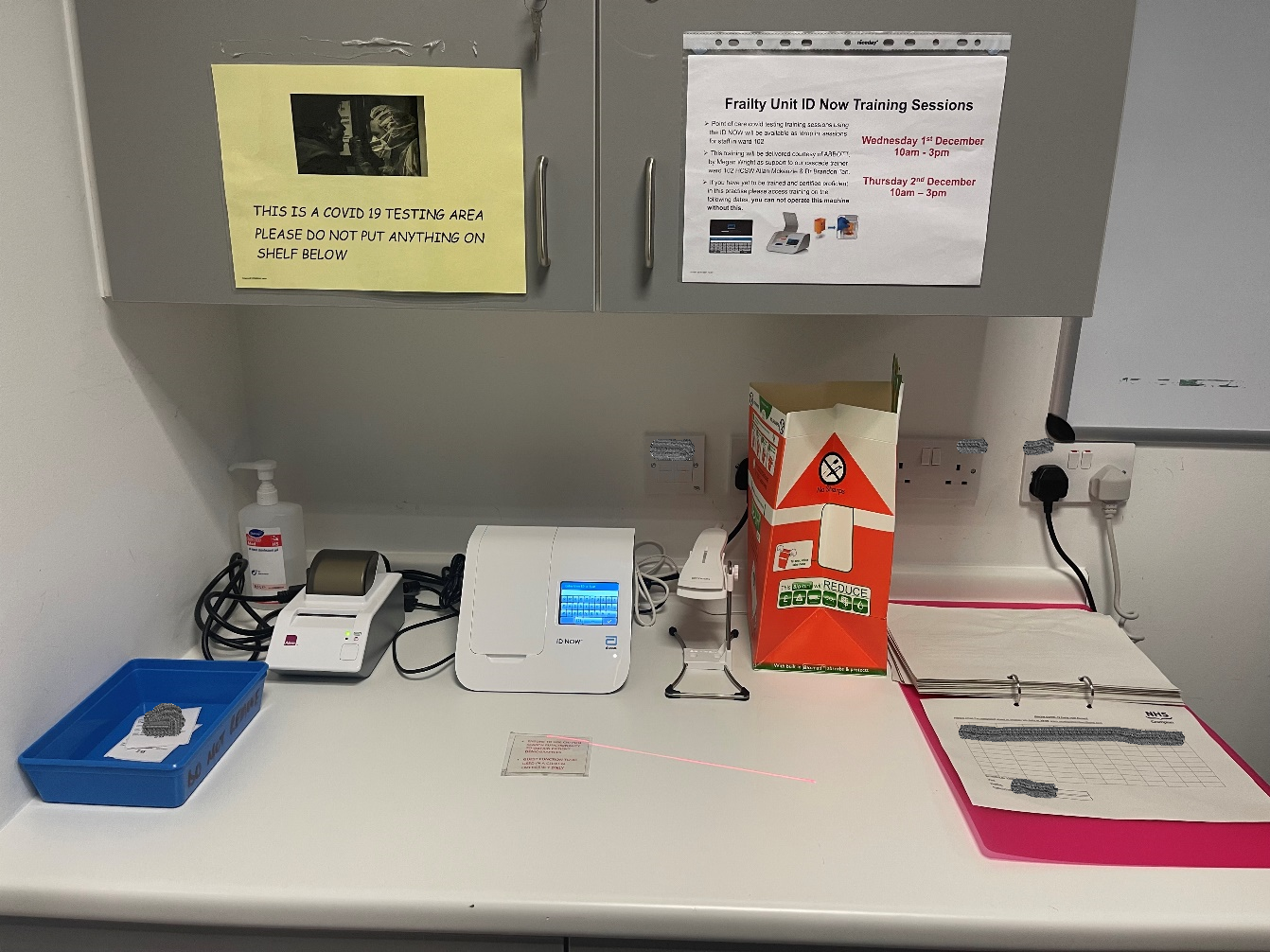

Supplement: S3 Fig — (TIF) [file pone.0296294.s003.tif]

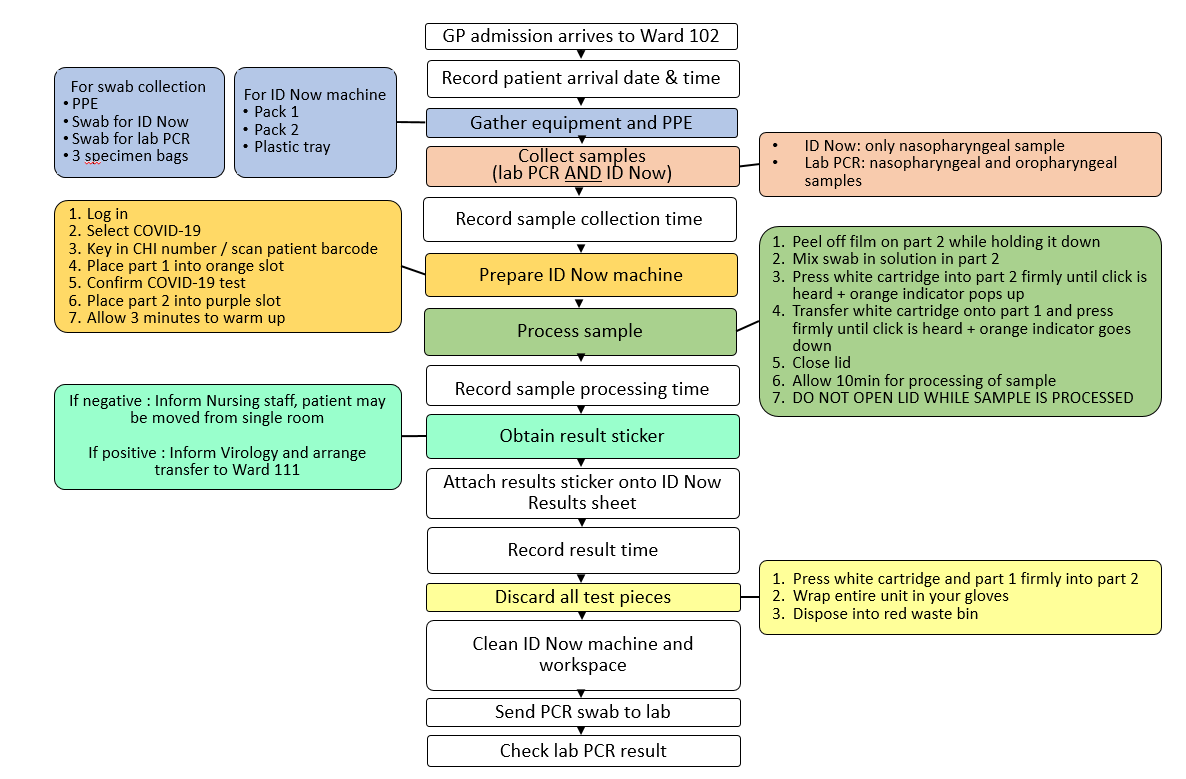

Supplement: S4 Fig — (TIF) [file pone.0296294.s004.tif]

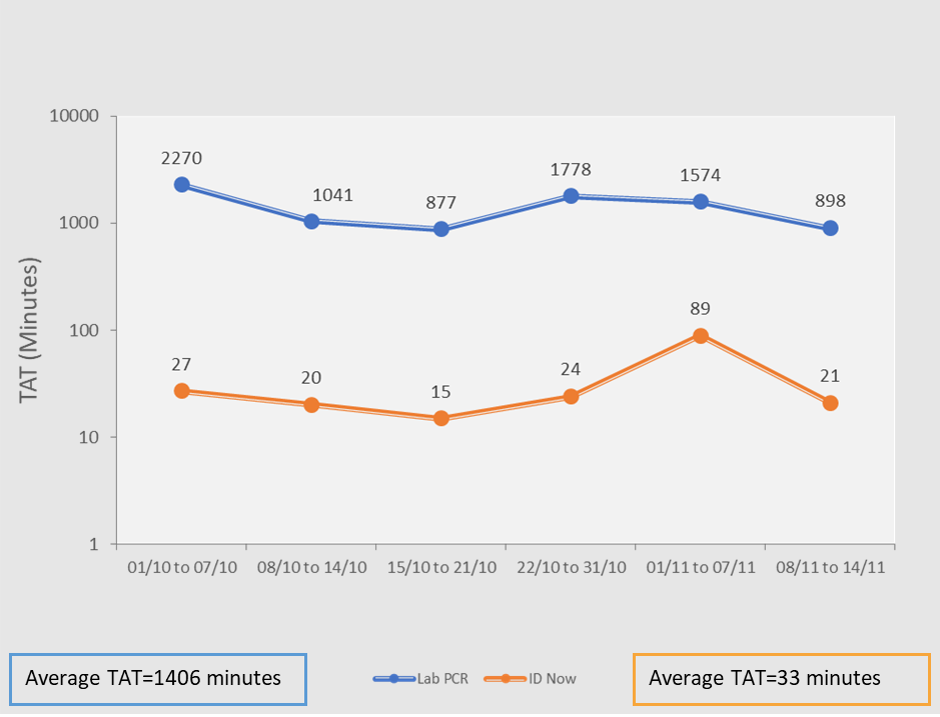

Supplement: S5 Fig — (TIF) [file pone.0296294.s005.tif]

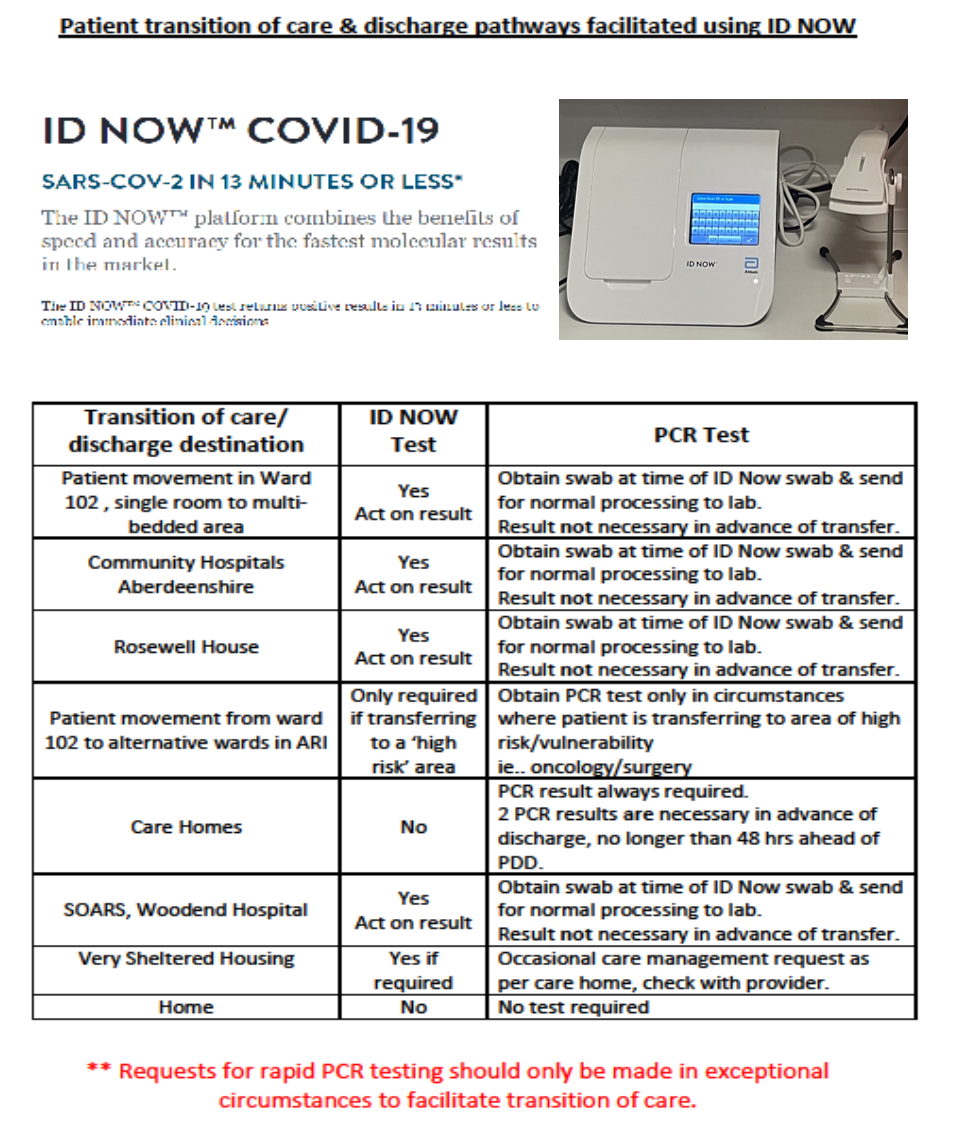

Supplement: S6 Fig — (TIF) [file pone.0296294.s006.tif]

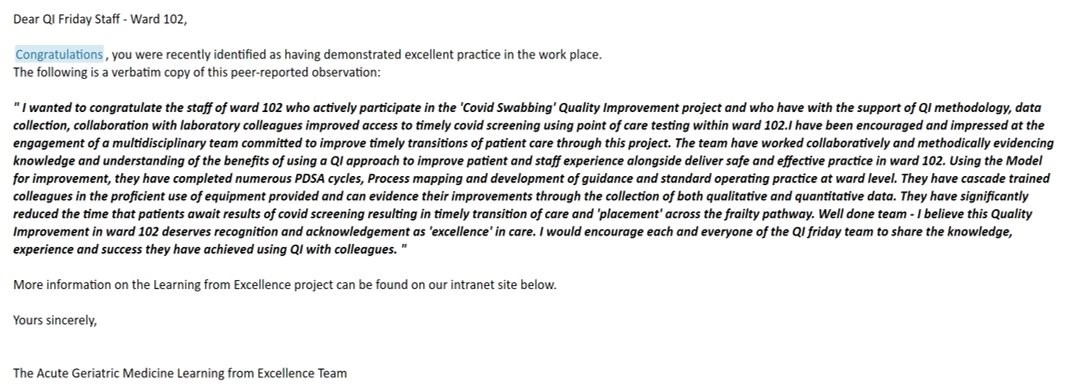

Supplement: S7 Fig — (TIF) [file pone.0296294.s007.tif]
